# High percentages of embryos with 21, 18 or 13 trisomy are related to advanced paternal age in donor egg cycles

**DOI:** 10.5935/1518-0557.20180004

**Published:** 2018

**Authors:** Javier García-Ferreyra, Roly Hilario, Julio Dueñas

**Affiliations:** 1 FERTILAB Laboratory of Assisted Reproduction, Lima, Peru; 2 PROCREAR Fertility Center, Lima, Peru; 3 Laboratory of Assisted Reproduction. Alcívar Hospital, Guayaquil, Ecuador

**Keywords:** aging, oocyte, aneuploidy, trisomy, PGD, ART

## Abstract

**Objective:**

Advanced paternal age is related to poor sperm quality; however, little is known on its effect on aneuploidy embryo rates and, more importantly, on chromosomal abnormalities like trisomy 21, 18 and 13. The objective of this study was to evaluate the effect of advanced paternal age on the trisomy rates of the chromosomes 21, 18 or 13 in embryos obtained from donated oocytes.

**Methods:**

A total of 378 embryos, obtained from 52 IVF/ICSI cycles with donated oocytes in conjunction with PGD, were allocated according to paternal age in three groups: Group A: ≤39 years (*n*=115 embryos), Group B: 40-49 years (*n=*157 embryos) and Group C: ≥50 year (*n=*106 embryos). Fertilization rates, embryo quality at day 3, blastocysts development, and aneuploidy embryo rates were then compared.

**Results:**

There was no difference in seminal parameters (volume, concentration and motility) in the studied groups. Fertilization rate, percentages of zygotes that underwent cleavage, and good-quality embryos on Day 3 were similar between the three groups evaluated. The group of men ≥50 years had significantly more sperm with damaged DNA, higher global aneuploidy rates, and significantly more embryos with trisomy 21, 18 or 13 compared to the other two evaluated groups (*p<0.05*).

**Conclusion:**

Our data shows that advanced paternal age increases global chromosomal abnormalities, and percentages of trisomy 21, 18 or 13 in embryos, and such effect is significantly important as of the age of 50. Embryo genetic screening is highly recommended in patients in which paternal age is ≥50 years old.

## INTRODUCTION

Assisted reproduction technologies (ART) enable treating most infertile couples in the world. Achieving satisfactory pregnancy and implantation rates, and the noninvasive selection of embryos free of chromosomal abnormalities is the main challenge in this human reproductive field. Aneuploidies are common in early human embryos (Munné & Cohen, 1998), and the most important contributor to poor outcomes in IVF. Their frequency in human preimplantation embryos generated during IVF is estimated to be between 56-84% (Fragouli *et al*., 2014), and their occurrence is related to maternal and paternal factors. Most aneuploidies are incompatible with life and result in non-viable embryos, manifested as developmental arrest prior to implantation, miscarriage or stillbirth. However, depending on the chromosome involved, some aneuploidies can be viable, such as trisomy 21 (Down syndrome), trisomy 18 (Edwards syndrome) and trisomy 13 (Patau syndrome). 

Down syndrome or trisomy 21, described in 1866 by John Langdon Down, is the most common neurodevelopmental disorder with a known genetic cause. It has a birth prevalence of 1:700-1:1000 live births (Savage *et al*., 1998), and its risk increases exponentially with increasing maternal age (Weijerman & de Winter, 2010; Oster-Granite *et al*., 2011). Approximately 95% of trisomy 21 babies happen by a maternal nondisjunction during the meiotic division, a 4% are due to a parental balanced robertsonian translocation between chromosomes 13 or 14 and 21; and 1% are caused by postzygotic mitotic nondisjunction (Gilbert-Barness, 2007). Trisomy 18, described by John Hilton Edwards in 1960, occurs in 1/7000 births (Goldstein & Nielsen, 1988), has a high percentage of prenatal fetal loss, and postnatally 60% of cases die within 2 months and more than 95% within a year. Most trisomy 18 cases are due to a maternal meiotic nondisjunction, with only 5% being caused by a parental balanced reciprocal translocation (Witters *et al*., 2011). Finally, trisomy 13, discovered by Klaus Patau in 1960, has an incidence of 1/19,000 live births (Goldstein & Nielsen, 1988), fetal loss around 97% and, in the postnatal period, nearly all trisomy newborns die within 4 months. The common cause of trisomy 13 is maternal meiotic nondisjunction (Witters *et al*., 2011). 

García-Ferreyra *et al*. (2015) evaluated the effects of male aging on aneuploidy rates in embryos obtained from donated oocytes, and reported that men ≥50 years significantly produced more aneuploidy embryos, and this event was related to high percentages of sperm DNA fragmentation. Moreover, regarding this association between advanced paternal age and risk of trisomy 21, 18 and 13, the studies are controversial. Studies by McIntosh *et al*. (1995) reported a 2-fold higher risk for trisomy 21 among fathers aged >50 years, compared to fathers of 25-29 years, after adjusting for maternal age and other factors. Similar results were also reported by Matsunaga *et al*. (1978) and Stene *et al*. (1981). Nevertheless, there is no evidence concerning the effects of advanced paternal age on trisomy 21 (Regal *et al*., 1980; Hook *et al*., 1981; Roth *et al*., 1983; Ferguson-Smith & Yates, 1984; Hook & Regal, 1984; Hatch *et al*., 1990; de Michelena *et al*., 1993). In relation to trisomy 18, studies carried out by Ferguson-Smith & Yates (1984), Hatch *et al*. (1990) and Naguib *et al*. (1999) did not show a paternal age effect on Edward's syndrome. Finally, Bugge *et al*. (2007) reported a greater paternal age in trisomy 13 cases with paternal meiotic origin compared to controls, but Hatch *et al*. (1990) did not find an association between male aging and trisomy 13 in spontaneous abortions. 

On the other hand, oocyte donation in which the effect of maternal age on oocyte and subsequent embryo is controlled provides an optimal model to study the influence of male aging on embryo quality and its implantation rate (Wong *et al*., 1996). Several studies have used this model obtaining high pregnancy rates and good obstetrical outcomes in recipients (Sauer & Kavic, 2006; Budak *et al*., 2007). Regarding chromosome abnormalities in embryos of egg donors, several studies reveal percentages of aneuploidy embryos to be between 39-82% (Reis Soares *et al*., 2003; Munné *et al*., 2006; Sills *et al*., 2014; García-Ferreyra *et al*., 2015; Haddad *et al*., 2015; Munné *et al.*, 2017), and only García-Ferreyra *et al*. (2015) evaluated the effects of paternal age on the aneuploidy.

The objective of this study was to evaluate the effects of advanced paternal age on the trisomy rates of the chromosomes 21, 18 and 13 in embryos obtained from donated oocytes.

## MATERIALS AND METHODS

### Study design

This is a retrospective nonrandomized study based on data analysis from 363 embryos, which were obtained from 50 IVF/ICSI donor egg cycles in conjunction with PGD (IVF: *n*=30; ICSI: *n*=22). In our center, all patients (including recipient of donated oocytes) are offered aneuploidy screening as a means to increase pregnancy rates, decrease loss rates, and avoid children with serious medical problems (e.g., Down syndrome). The procedures were done at FERTILAB Laboratory of Assisted Reproduction (Lima, Peru) between January 2012 and July 2016. Written informed consents were obtained from all recipients and their partners included in this study to share the outcomes of their cycles for research purposes. This study was approved by the Institutional Review Board and the corresponding Ethics Committee from Clínica Oncogyn (Lima, Peru).

Thirty-four anonymous oocyte donors (20-30 years old) underwent physical, gynecological and psychological examinations and there were no family histories of hereditary or chromosomal diseases. All participants had a normal karyotype and tested negative for sexually transmitted diseases. Oocyte donor Recruitment was done based on recommendations provided by other donors, and the donation of their gametes was merely for altruistic reasons.

### Controlled ovarian stimulation and oocyte collection

The menstrual cycles of oocytes donors were stimulated using recombinant FSH (rFSH) (Gonal^®^, Merck Serono laboratories, Peru) according to previously established stimulation protocols (Tavmergen *et al*., 2002). Medication was started on day 2 of the menstrual cycle until at least three follicles reached ~18 mm in diameter. The oocytes were collected 36h after hCG administration (Pregnyl^®^, Ferring Farmaceutical, Peru) by transvaginal ultrasound ovum pick-up. During the follicular aspiration procedure, the oocytes were recovered in Global^®^-HEPES-buffered medium (LifeGlobal) supplemented with 10% vol/vol Serum Substitute Supplement (SSS; Irvine Scientific). After retrieval, cumulus-oocyte complexes were trimmed of excess cumulus cells using sterile needles and cultured in ~200 µL drops of Global^®^-Fertilization medium (LifeGlobal) plus 10% SSS under oil at 37°C and room air containing 6% CO_2_, 5% O_2_ and 89% N_2_ for 5 hours before the IVF/ICSI procedure.

### Insemination, fertilization and embryo culture

The recovered oocytes were assessed for their nuclear maturity, and only metaphase II oocytes were submitted to IVF/ICSI. Insemination was made with 50,000-100,000 motile spermatozoa in ~200 µL drops of Global^®^-Fertilization medium + 10% SSS, where 1 to 5 oocytes were placed. In the ICSI procedures, all collected oocytes were enzymatically denuded off cumulus cells using hyaluronidase (80 IU/mL; LifeGlobal), and injected following routine procedures (García *et al*., 2007).

Normal fertilization was evaluated 16-18 hours after insemination/injection by the presence of two pronuclei (day 1). The zygotes were individually cultured in mineral oil, in 10-µL droplets of Global^®^ medium (LifeGlobal) supplemented with 10% vol/vol SSS from day 1 to day 3, in which the embryos were moved to fresh Global^®^ medium and cultured for 2 days more up to blastocyst stage. On day 3 the embryos were evaluated for cell number, fragmentation and multinucleation and, on day 5, for development to blastocyst and expansion. Good quality day-3 embryos were defined as those with 6-8 cells and ≤10% of fragmentation. Good quality blastocysts were defined as having an inner cell mass (ICM) and type A or B trophectoderm (Gardner & Schoolcraft, 1999).

### Embryo biopsy, fixation and FISH analysis

On the third day after insemination, one cell per embryo was biopsied following a protocol described elsewhere (Munné *et al*., 2003). Individual embryos were placed into calcium/magnesium-free media (PGD Biopsy Medium; LifeGlobal), through a hole on the zona pellucida, opened with Tyrode's acid solution; one nucleated blastomere was removed by aspiration. After biopsies, the embryos were rinsed thoroughly and returned to culture in mineral oil, in 10-µL droplets of Global^®^ medium (LifeGlobal) supplemented with 10% vol/vol SSS.

Blastomeres were fixed individually following routine protocols to minimize signal overlap and loss of micronuclei (Velilla *et al*., 2002). PGD analysis was performed by FISH, using probes specific for twelve chromosomes 8, 13, 14, 16, 18, 20, 21, 22 (Abbott Laboratories), X, Y, 15 and 17 (Cellay Inc) following the manufacturer's instructions.

### Sperm collection

Semen samples were collected by masturbation after 3 days of abstinence and on the day of oocyte retrieval. Semen analysis was performed according to World Health Organization criteria (WHO, 2010). After liquefaction, motile spermatozoa were separated from the seminal plasma by centrifugation at 300 X *g* for 10 minutes through 1.0 mL 95% and 45% isolate gradients (Irvine Scientific). The pellet was washed once by centrifugation for 5 min, and was resuspended in 0.1 mL of Global Fertilization medium + 10% SSS for IVF/ICSI.

### Sperm DNA fragmentation assessment

Prior to the hormonal stimulation, sperm DNA fragmentation values were evaluated with the Sperm Chromatin Dispersion (SCD) test (Fernández *et al*., 2003) using the Halosperm^®^ Kit (Halotech DNA, Spain). Operators scored ≥500 spermatozoa for each patient according to the patterns established by Fernández *et al*. (2003). Sperm nuclei with fragmented DNA produced very small or no halos of dispersed DNA at all, and nuclei without DNA fragmentation released their DNA loops to form large halos.

### Statistical Analysis

The data was statistically analyzed using the χ^2^ test and Student's t-test as appropriate, and differences were considered to be significant at *p<*0.05. All statistical analysis was carried out using the statistic package Stata 10 (StataCorp, College Station, TX, USA).

## RESULTS

Results of chromosomal status from 378 biopsied embryos were allocated to three groups according to paternal age:

- ≤39 years (range 30-39 years; *n*=16)
- 40-49 years (range 40-48 years; *n*=22)
- ≥50 years (range 50-68 years; *n*=14)

There was no difference in oocyte donor age (24.2±2.21, 24.7±2.11 and 24.2±2.58 years), days of stimulation (8.7±1.05, 8.8±1.08 and 8.6±0.94) and mean of rFSH treatment (1295.1±291.58, 1334.6±250.54 and 1299.1±93.62 IU/L) between the three evaluated groups (data not shown). 

Semen characteristics and sperm DNA fragmentation according to male age are shown in Table 1. Values of semen volume, sperm concentration and progressive motility were similar in the three evaluated groups (*p*=not significant). Men ≥50 years old had significantly less spermatozoa with normal morphology, compared to men ≤39 years old (5.5±4.70% versus 9.3±5.44%; *p<0.05*). Likewise, patients ≥50 years old had significantly high percentages of sperm with fragmented DNA (33.6±18.19% versus 25.6±15.63% and 24.1±14.49%) compared to the groups ≤39 years old and 40-49 years old respectively (*p<0.05*). 

**Table 1:** Comparison of seminal characteristics according the male age

	**≤39**	**40-49**	**≥50**
Semen volume (mL) (Mean ± SD)	2.1±1.19	1.9±1.31	1.9±1.10
Sperm concentration (x 10^6^/mL)	125.4±46.13	95.4±43.66	56.8±50.69
Progressive motility (%)	30.3±7.48	28.1±8.55	25.2±9.07
Sperm morphology (%)	9.3±5.44	7.4±3.84	5.5±4.70*
Sperm DNA fragmentation (%)	25.6±15.63	24.1±14.49	****33.6±18.19**

* *p<0.05* in relation to groups ≤39 years

** *p<0.05* in relation to groups ≤39 years and 40-49 years

A total of 175, 261 and 155 oocytes were inseminated from ≤39 years, 40-49 years and ≥50 year's groups, respectively. Fertilization rate (2PN) (90.9%, 83.1% and 81.3%), percentages of zygotes that underwent cleavage (98.7%, 98.6% and 96.1%), mean cell number (7.3±1.08, 7.3±0.97 and 7.3±1.19), good-quality embryos on day 3 (77.7%, 78.9% and 82.6%), blastocyst formation rate (47.8%, 47.9% and 42.1%), and good-quality blastocysts (88.2%, 83.2% and 81.2%) were similar from ≤39 years, 40-49 years and ≥50 year's groups, respectively (Table 2).

**Table 2:** Comparison of laboratory results between the three evaluated groups

	**≤39**	**40-49**	**≥50**
No. total oocytes	193	294	174
No. total inseminated oocytes	175	261	155
No. total fertilized oocytes (2PN) (%)	159 (90.9)	217 (83.1)	126 (81.3)
No. total cleaved embryo on Day 3 (%)	157 (98.7)	214 (98.6)	121 (96.1)
No. cell/embryo on Day 3 (Mean ± SD)	7.3±1.08	7.3±0.97	7.3±1.19
Good-quality embryos on Day 3 (%)	77.7	78.9	82.6
Blastocyst formation/2PN (%)	47.8	47.9	42.1
Good-quality blastocysts (%)	88.2	83.2	81.2

**Figure 1:**
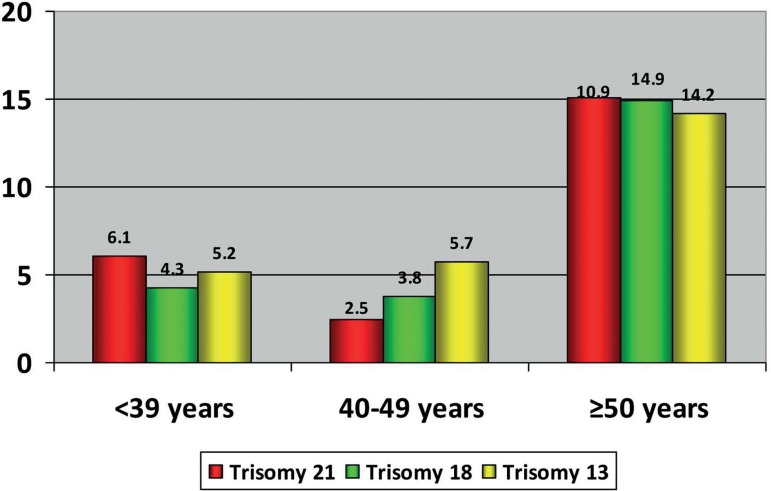
Percentage of embryos with trisomy 21, 18 and 13 in men of ≤39 years, 40-49 years and ≥50 years

**Figure 2:**
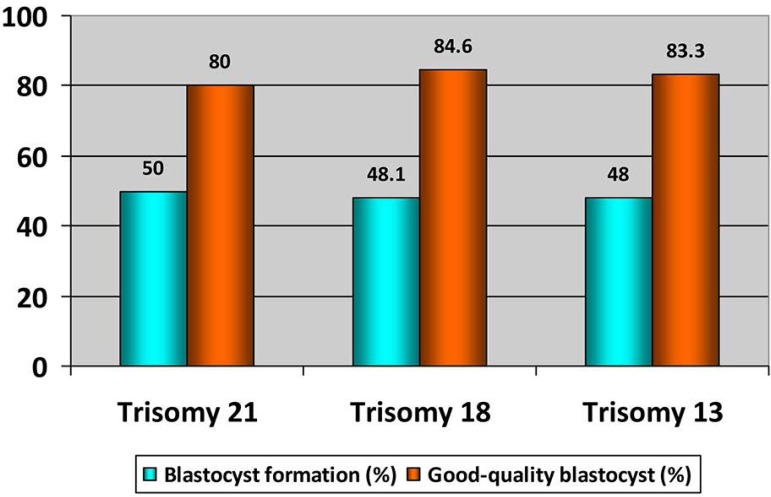
Blastocyst formation rate and Good-quality blastocyst in embryos with trisomy 21, 18 and 13

The characteristics of chromosome abnormalities in the three evaluated groups are summarized in Table 3. Advanced paternal age was significantly associated to high aneuploidy rates in embryos; thus 65.1% of the embryos from the group of ≥50 years old were aneuploidies compared to 55.6% in the group of ≤39 years and 53.5% in the group of 40-49 years old (*p<0.05*). Similarly, the prevalence of embryos with trisomy 21, 18 or 13 was significantly higher in older men (≥50 years old) than that observed in the other evaluated groups (Trisomy 21: 15.1% versus 6.1% and 5.7%; Trisomy 18: 14.9% versus 4.3% and 3.8%; Trisomy 13: 14.2% versus 5.2% and 2.5%; *p<0.05*) (Figure 1). From embryos with any trisomy, 50% (trisomy 21), 48.1% (trisomy 18) and 48% (trisomy 21) achieved the blastocyst stage. Additionally, good quality blastocysts were similar in the three trisomy types (Trisomy 21: 80%, trisomy 18: 84.6%, and trisomy 21: 83.3%) (Table 4 and Figure 2).

**Table 3:** Total aneuploidy rate and percentages of embryos with trisomy 21, 18 and 13 in men of ≤39 years, 40-49 years and ≥50 years

	**≤39**	**40-49**	**≥50**
No. total embryos biopsied/2PN (%)	115 (72.3)	157 (72.4)	106 (84.1)
Total aneuploidy rate (%)	55.6	53.5	65.1*
Embryos w/trisomy 21 (%)	6.1	5.7	15.1*
Embryos w/trisomy 18 (%)	4.3	3.8	14.9*
Embryos w/trisomy 13 (%)	5.2	2.5	14.2*

* *p*<0.05 in relation to groups ≤39 years and 40-49 years of age

**Table 4:** Blastocyst formation rate (BFR) and Good-quality blastocyst (GQB) in embryos with trisomy 21, 18 and 13

	**Trisomy 21**	**Trisomy 18**	**Trisomy 13**
Blastocyst formation rate (%)	50.0	48.1	48.0
Good-quality blastocyst (%)	80.0	84.6	83.3
Full blastocyst (%)	46.6	15.4	16.7
Expanded blastocyst (%)	26.7	23.1	25.0
Hatching blastocyst (%)	26.7	61.5	58.3

## DISCUSSION

In the present study, we evaluated the effects of male age on aneuploidy embryo rate and prevalence of trisomy 21, 18, or 13 including only IVF/ICSI cycles using donor oocyte for controlling female age. The data obtained demonstrates a significant negative effect of paternal age beginning from ≥50 years on the aneuploidy, increasing the percentages of embryos with trisomy 21, 18, or 13. Our results are very important because they directly show that the number of trisomy embryos is higher with paternal aging when the effect of the female age is controlled.

Aneuploidies are the principal cause of implantation failures and miscarriages in human reproduction. Some chromosome anomalies can be compatible with life (trisomy 21, 18 and 13), but still produce severe problems in intrauterine and neonatal development, resulting in premature natal death. Down babies have facial dysmorphism with a flattened skull, an enlarged tongue, epicanthal folding, brush field spots (small white spots) in the iris and small low-set ears with a prominent overlapping anti-helix, respiratory disorders, congenital defects of the gastrointestinal tract, endocrine and urinary tract (Roizen & Patterson, 2003). Trisomy 18 has typical phenotypes, including facial dysmorphism with micrognathia, low set abnormal ears, hirsutism, spina bifida, omphalocele, heart defects, clubfeet, and radial aplasia (Gilbert-Barness, 2007; Witters *et al*., 2005). Finally, trisomy 13 is characterized by a reduced fetal size for gestational age, central nervous system anomalies, holoprosencephaly, midline facial defects, spina bifida, and urogenital malformations (Gilbert-Barness, 2007; Goetzinger *et al*., 2008). 

There is increasing evidence indicating that advanced paternal age causes several changes in reproductive functions, including decreased circulation of androgens (Kaufman & Vermeulen, 2005), elevated levels of FSH (Johnson *et al*., 1990), alteration of testicular morphology (Dakouane *et al*., 2005), changes in spermatozoa production and characteristics (Kühnert & Nieschlag, 2004; Hellstrom *et al*., 2006; Sloter *et al.*, 2006), and a significant increase in spermatic DNA fragmentation in men >50 years of age (García-Ferreyra *et al*., 2012; 2015), similar to the results observed in the present study. These age-related paternal factors may increase the rates of embryonic aneuploidies and lead to miscarriages (de la Rochebrochard & Thonneau, 2002; Nybo Andersen *et al*., 2004), congenital birth defects (Olshan *et al*., 1995; Tellier *et al*., 1998) lead to the development of several syndromes, such as achondroplasia and Apert (Crow, 2000), neurological disorders (Malaspina *et al*., 2002), and some cancers (Zhang *et al*., 1999). It is likely that these abnormalities are due to the inability to repair DNA and/or high levels of meiotic errors (Sartorius & Nieschlag, 2010).

High percentages of spermatozoa from subfertile and infertile men have damaged DNA; being apoptosis, abnormal chromatin packaging, and reactive oxygen species, the principal molecular mechanisms leading to DNA fragmentation (Sakkas *et al*., 1999). Several studies have shown that spermatozoa with fragmented DNA are able to fertilize an oocyte, but may result in poor quality embryos, blastocyst development blockage, high aneuploidy rates in embryos, and lower pregnancy and implantation rates in IUI, IVF, or ICSI procedures (Larson *et al*., 2000; Duran *et al*., 2002; Henkel *et al*., 2004; Muriel *et al*., 2006; García-Ferreyra *et al*., 2015). Our study reveals an age-dependent increase in sperm DNA fragmentation, which is statistically significant in males ≥50 years old. Similar results were previously reported by Plastira *et al*. (2007), Vagnini *et al*. (2007) and García-Ferreyra *et al*. (2012). Likewise, advanced paternal age was related to high total embryonic aneuploidy rates and embryos with trisomy 21, 18, or 13, indicating that it is very important to recommend genetic screening of embryos to patients when the male is over 50 years of age. It has been well documented that advanced paternal age is associated with reduced fertility and higher risks of miscarriage (de la Rochebrochard & Thonneau, 2002; Kleinhaus *et al*., 2006), but its effect on the development of trisomy 21, 18 and 13 is still controversial. For example, in relation to trisomy 21, some authors have indicated advanced paternal age to be crucial (Erickson & Bjerkedal, 1981; Stene *et al*., 1981; Thepot *et al*., 1993; McIntosh *et al*., 1995; Oliver *et al*., 2009), while others saw no association (Regal *et al*., 1980; Hook *et al*., 1981; Roth *et al*., 1983; Ferguson-Smith & Yates, 1984; Hook & Regal, 1984; Hatch *et al*., 1990; de Michelena *et al*., 1993). These contradictory observations may be a result of studying trisomy risk in offspring without taking into consideration trisomy embryos that were not implanted or were eliminated early after implantation. Subsequently, a misdiagnosis about the real effect of advance paternal age on chromosomal diseases was carried out. 

Most aneuploidies found in embryos originate from the oocyte; however, in the last years several studies have showed an effect of advanced paternal age on aneuploidy rates (Magli *et al*., 2009; García-Ferreyra *et al*., 2015), and at least fivefold higher risk of miscarriages (de la Rochebrochard & Thonneau, 2002). Aneuploidy in embryos can originate by changes in the centrosome that result in abnormal spindle formation and chromosome malsegregation (Palermo *et al*., 1994), or by the generation of aneuploid gametes during spermatogenesis; and patients with oligoasthenoteratospermia or nonobstructive azoospermia (testicular sperm extracted) with severe defects that may result in higher percentages of mitotic abnormalities and chaotic embryos (Magli *et al.*, 2009). On the other hand, the paternal effect on embryo development can be evaluated after the eight-cell stage when embryo genome activation occurs and thus, paternal genetic information is expressed (late paternal effect) (Tesarik, 2005). This effect causes blastocyst formation failure and low clinical outcomes, and is related to male aging and high percentages of sperm DNA fragmentation (García-Ferreyra *et al*., 2015). In the present study, there were no differences in fertilization rates, and embryo quality until day 3 in the three age groups, similar to previously reported results (Gallardo *et al*., 1996; Frattarelli *et al*., 2008; Ferreira *et al*., 2010). During culture extended to day 5, a reduction in the blastocyst formation rate in the group of males ≥50 years of age was observed, compared to the other evaluated groups, but this difference was not significant. Furthermore, it is important to highlight that about half of the embryos with some trisomy reached the blastocyst stage, and around 80% of them were good-quality embryos, which increases the possibility of normal implantation rates and subsequent miscarriage or the birth of a child with genetic disorders. Our findings are very important because they demonstrate that advanced paternal age actively contributes to a higher rate of chromosomal abnormalities in the resulting embryos, and when the type or extent of DNA damage cannot be balanced by the reparative ability of the oocyte (including cases of egg donor), then the genetic screening in embryos should be considered to improve clinical outcomes. Egg donor programs can be considered successful largely because oocyte quality is greatly improved when the donor's age is low and by the absence or minimal chromosomal errors in the oocytes. However, Battaglia *et al*. (1996) showed that 17% of human egg collected from healthy women at the age of 22-25 years during natural cycle had spindle abnormalities, which cause aneuploidy in embryos derived from donated oocytes. In the present study, we report a global aneuploidy rate of 57.4%, similar to what was demonstrated by Sills *et al*. (2014), Haddad *et al*. (2015) and García-Ferreyra *et al*. (2015), but this percentage increased to 65.1% when the male patient was ≥50 years old, suggesting that this event is related to high values of sperm DNA fragmentation. On the other hand, advanced paternal age affects the genetic characteristics of embryos, including those obtained from donated oocytes.

In conclusion, our data shows that male aging increases chromosomal abnormalities in the resulting embryos from egg donor, being this effect significant from the age of 50 and older. Genetic screening in embryos should be recommended in egg donor cycles and especially if paternal age is ≥50 years in order to obtain better clinical outcome and reduce the likelihood of abnormal pregnancies, that may end in spontaneous abortions, intrauterine fetal death, intrauterine growth retardation or offspring with several congenital defects.
